# “Wonderful! We’ve just missed the bus.” – Parental use of irony and children’s irony comprehension

**DOI:** 10.1371/journal.pone.0228538

**Published:** 2020-02-21

**Authors:** Natalia Banasik-Jemielniak, Sandra Bosacki, Anna Mitrowska, Diana Wyrębek Walters, Katarzyna Wisiecka, Natalia Ewelina Copeland, Lara Wieland, Ljiljana Popovic, Jovana Piper, Aleksandra Siemieniuk

**Affiliations:** 1 Institute of Psychology, The Maria Grzegorzewska University, Warsaw, Poland; 2 Faculty of Psychology, University of Warsaw, Warsaw, Poland; 3 Department of Educational Studies, Brock University, St. Catharines, Canada; 4 Faculty of Psychology, SWPS University of Social Sciences and Humanities, Warsaw, Poland; 5 Faculty of "Artes Liberales", University of Warsaw, Warsaw, Poland; 6 Einstein Center for Neurosciences Berlin, Charité Universitätsmedizin Berlin, Berlin, Germany; 7 Faculty of Philosophy, University of Belgrade, Belgrade, Serbia; Middlesex University, UNITED KINGDOM

## Abstract

Irony is one of the linguistic means in which intended and expressed meaning diverge. It serves social-communicative functions, requires the understanding of the speaker's mental state and its comprehension takes place at an advanced stage of language acquisition. In the present study, we investigated 8-year old’s irony comprehension and social skills and asked their parents about their preferred use of irony towards their children. We then compared children with the highest scores in irony comprehension test with those with lower scores. The full sample included 46 families from Poland. Results show positive associations between children’s levels of irony comprehension and levels of mothers irony use. No such relations were found for fathers. No differences were found in ToM scores between proficient and non-proficient irony comprehenders. Our findings provide a base for future studies to study the use of irony in child-parent talk in more diverse culturally and linguistically diverse populations.

## 1. Introduction

Traditionally, irony is described as a situation when a speaker says X while meaning non-X [1–4], for instance, saying: “Great! This is just wonderful” in a situation when something goes wrong, uttered to express disappointment with what is happening. In Grice’s view, figurative meanings such as irony are implicatures, which are based on a violation of the first maxim of quality (do not say what you believe to be false) [5] In the neo-Gricean perspective, it is stressed that irony inherently carries implicit evaluation, feeling or an attitude [6].

Although development of irony production mainly takes place in adolescence [7]. comprehension of irony emerges much earlier in childhood and develops throughout adolescence and adulthood. Irony comprehension has been considered to be a skill that emerges relatively late in development, even in comparison with other forms of figurative language [8]. In several studies, it has been shown that 6-year-olds understand the discrepancy between the surface and the intended meaning in a spoken ironic utterance [9–14], as well as the intentions of the speaker [15]. They can also detect some pragmatic functions of irony [e.g.^8^, ^12^, ^14^ ^16^] Some studies reported frequent failures with children as late as when they are 13 [17–18]. However, results from some newer studies show that this emerging skill can be observed much sooner, even with 3-and 4-year-olds [19–22]. The understanding of irony requires a correct identification of the speaker's intention and the understanding of the attitude towards the situation or person the comment refers to [23].

This links the ability to comprehend figurative meanings to social skills, such inferring about other people’s mental states. Through irony, the speaker conveys beliefs and attitudes in an indirect way and the listener needs to substitute the literal meaning with the intended, implied one.

Several factors have been described as correlates of irony understanding (e.g., how accurately it is interpreted, and what pragmatic impact it has on the listener) and use (e.g., how often and in what contexts is the speaker likely to use it) in adults. Among others, these include gender [24] and social competences [25] Additionally, research on irony in various languages shows different results about the role of lexical markers and acoustic parameters [26]. Acoustic markers for irony also differ across languages [27]. Finally, not only the individual personalities of the interlocutors, but also their wider social context can play an important role in irony use and understanding.

Taking the theoretical perspective of sociocultural learning, which is based on the concept that human activities take place in a cultural context and that social interaction plays a fundamental role in the development of cognition, we are looking at children’s comprehension of irony in the family context [28].

It is well established that family environment plays a crucial role for the development of the child’s language skills [29–32], as well as social skills [31, 33–36]. However, little is known about parent-child talk in the perspective of the development of children’s pragmatic elements of language, such as the ability to comprehend figurative speech, especially irony [37, 38].

Although an increasing number of studies explore the development of irony in children [21, 19, 39, 40], to the best of our knowledge, to date there is no study that specifically investigated if children with richer ironic input from parents comprehended irony to a larger extent than children who receive less ironic input.

Parents’ beliefs and attitudes towards linguistic and non-linguistic behaviors are related to their actual behaviors, as well as to the children’s outcomes in relation to these behaviors [41, 42]. Hence the assumption that parents’ declared frequency of ironic comments directed to their children is related with the actual irony use in parent-child conversations, seems warranted.

The tendency for parents to use irony in conversations with their children may be crucial in the development of irony comprehension as it is an important part of social development [9, 23, 14]. Irony comprehension involves a ‘thought about a thought,’ and consequently, requires a more complex form of mind reading ability than other examples of figurative language such as metaphors [43]. Further, [44] found that children’s second-order higher order false belief reasoning or the ability to understand another person's belief about another person's belief (e.g., R thought that S thought R knew that…) predicted understanding of irony. These results highlight that to help children interpret their parents’ use of irony in day to day conversations they need to develop higher mental processing abilities such as metacognitive and mindreading abilities.

## 2. The present study

To the best of our knowledge, to date, little research exists on the frequency of parents’ use of irony in parent-child talk, and how parental use of irony influences their children’s irony comprehension. In addition, few studies explore the influence of gender in the link between parental use of irony and their children’s irony comprehension [22].

To address these gaps in the literature, the aim of the present study was to investigate whether a difference exists between children who exhibit high levels understanding of simple ironic utterances well, and children who show low understanding in terms of their parents’ irony use during their interactions with their children. Also, based on past studies that show positive links between children’s irony understanding and their social skills, particularly their ability to understand thoughts and emotions in others or Theory of Mind (ToM), we were interested in possible differences between the two groups (high and low irony comprehenders) and in their ToM [45].

Our research questions were as follows:

1. Do differences exist in parental uses of irony between children who exhibit high levels of irony understanding and children who show low levels of irony understanding?
2. Do relations exist between parents’ attitude towards irony and their children’s social skills such as ToM?
3. Does gender (of parent/child) affect these individual differences and relations among the variables?

## 3. Method

We have received the written permission from the IRP of Faculty of Psychology, University of Warsaw. No number was given to the permission.

### 3.1 Participants

Data was gleaned from a larger cross-cultural project on parent-child communication and children’s social cognitive development and understanding of linguistic indirectness. The sample included 46 families from Warsaw, Poland. Inclusion criteria for families were the presence of one 8-year old child (male or female) and non-separated, heterosexual parents. The inclusion criteria were meant to create a relatively homogenous sample, which would make it easier for us to infer about possible language input the children are receiving when analyzing the data on their irony comprehension.

The sample was homogenous in terms of SES, race and ethnicity: it included middle class, Caucasian parents of Polish background. The majority of the parents have completed higher education (university degree). Before starting the research, ethical clearance was received from University of Warsaw approving this project (WP UW, 2017). Parent written informed consent of all participating children was obtained.

Our sample includes 24 girls, 22 boys, 46 mothers and 41 fathers (four of the fathers in the families tested did not find time to schedule a meeting with the researcher). Children’s mean age was 8 years; 5 months (SD = 3.9) and all of them attended public school grade 2 at the time of testing. All children were monolingual speakers of Polish, which was verified by interviewing parents. The pre-interview phase included questions about any atypicalities in language or cognitive development, to include in the study only those children who were not diagnosed with any learning exceptionalities. All the families tested were included in the final analysis. The families were recruited through social media and snowballing.

### 3.2 Materials

The instruments used in the study were: 1) questionnaires and tasks used with children and 2) questionnaires used with parents.

1. Questionnaires and tasks used with children
  We present instruments in the same order that they were administered to children:
  *1.1* To measure higher order Theory of Mind in children [46] and social skills, we administered the *Social Ambiguous Stories Task* (SAS) [47]. This measure includes two short stories consisting of an ambiguous social situation with three characters. The stories were read to children while pictures accompanying the stories were presented to them on a computer screen. Participants were asked to answer questions regarding the thoughts, emotions and intentions of the characters [49] A coding scheme developed by Bosacki [47]was used to assess the child’s ability to interpret mental states. For each of the answers, 0 points were given for "I don't know", “no” answer or tangential responses, 1 point for responses that included behavioral or situational descriptions, 2 points for responses that included mental states or acts of communication or perception, and 3 points for responses that included an integration of two or more mental states and related them to each other in a coherent manner. The scoring measures 4 dimensions: comprehension of the story (2 questions, max. score of 2 points), conceptual role taking (4 questions, max. score of 12 points), emphatic sensitivity (4 questions, max score of 12 points), person perception (1 question, max. score of 2), alternative explanation (1 question, max. score of 3). This measure has been used with children and adolescents aged 6 to 16, and has been found to show relatively high levels of reliability and validity, with Cronbach’s alpha .67 for the task for girls and .69 for the task for boys [46–49].
  *1.2 Irony Comprehension Task–short version (ICT-sv)*, was obtained by shortening the Irony Comprehension Task by [19]. which showed a satisfactory external validity [50]. The shortened task is composed of three stories describing situations, in which one of the characters makes an ironic comment to a child. The stories are read by the researcher to the child while showing pictures on a computer screen. After each story, the child is asked a series of questions about the ironic comment: an open question ‘Why do you think X said Y?”, a comprehension question which was counted as a score of irony comprehension: “When X said Y, did they mean Y or Z?” of every character. The participants were then asked in two questions to assess how funny the ironic comment was (1) and how nice/mean the character uttering it was (2). For the score of comprehension, one question was scored for each of the presented stories, that is the total score of 3 could be obtained. The stories used can be found in attachment 1. The task has been shown to have appropriate content validity. Reliability of the measure was not tested.
2. Questionnaires used with parents
  *1.1 Attitude Towards Irony* (ATI), which presented different situations of parents using irony towards their child. Research participants had to decide whether they would be likely to respond as the examples in the exact situation. In close-ended questions the scale ranged from 0 to 5. Thus, 0 points were assigned for “never”, 1 point was for “maybe in some circumstances”, 2 points for “probably yes” and 5 points for “yes”. The reliability and the validity of the questionnaire were not tested.

### 3.3 Procedure

Participants were tested in their homes. Parents completed self-report questionnaires in one room, while the researcher tested the child in separate room within the house. Testing took approximately thirty minutes for children and forty minutes for parents and breaks were permitted according to the participant’s needs. The experimenter presented the child first with the Social Ambiguous Stories Task (used to assess ToM), followed by the Irony Comprehension Task. Parents completed a set of questionnaires and a survey on demographic information in a separate room. Before testing, participants were ensured that their responses are confidential. Also, they knew they could ask questions or stop testing at any moment.

### 3.4 Statistical analyses

We used SPPS IMAGO software to calculate the results. We first run a normality test looking at the distribution of the score for irony comprehension. To do this, we used the Shapiro-Wilk normality test. Since the significance of the test was 0.000m the hypothesis on the normality of distribution was rejected and hence we needed to use non-parametric tests instead of parametric ones. To compare two groups, we used U-Mann Whitney test.

## 4. Results

**Fig 1:**
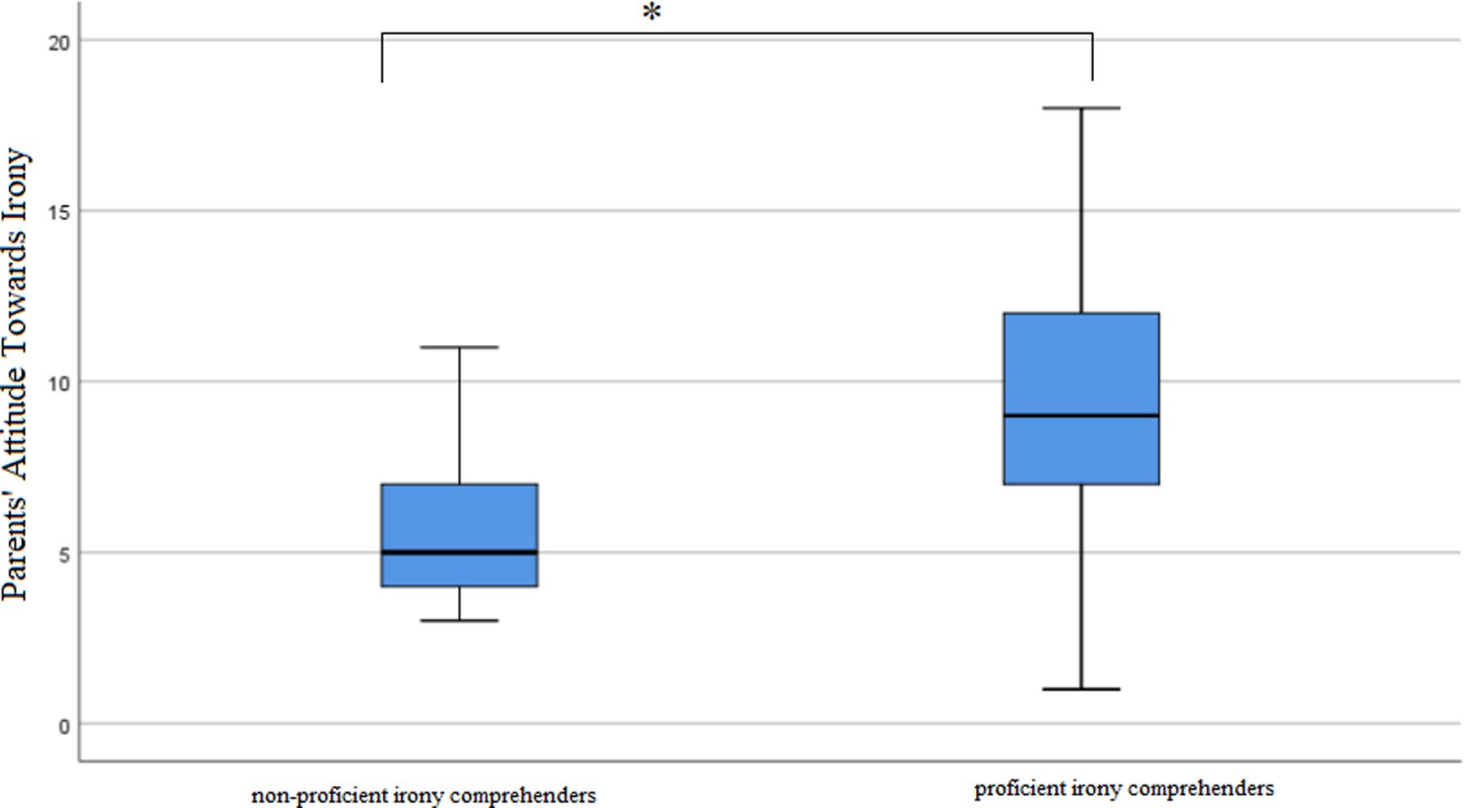
Mann-Whitney U test results displayed in box plot showing significant differences in parents’ declared likelihood of using irony between a group of children not proficient and proficient in decoding the right meaning behind the ironic statement (*U* = 72.5, *p* = 0.016).

**Fig 2:**
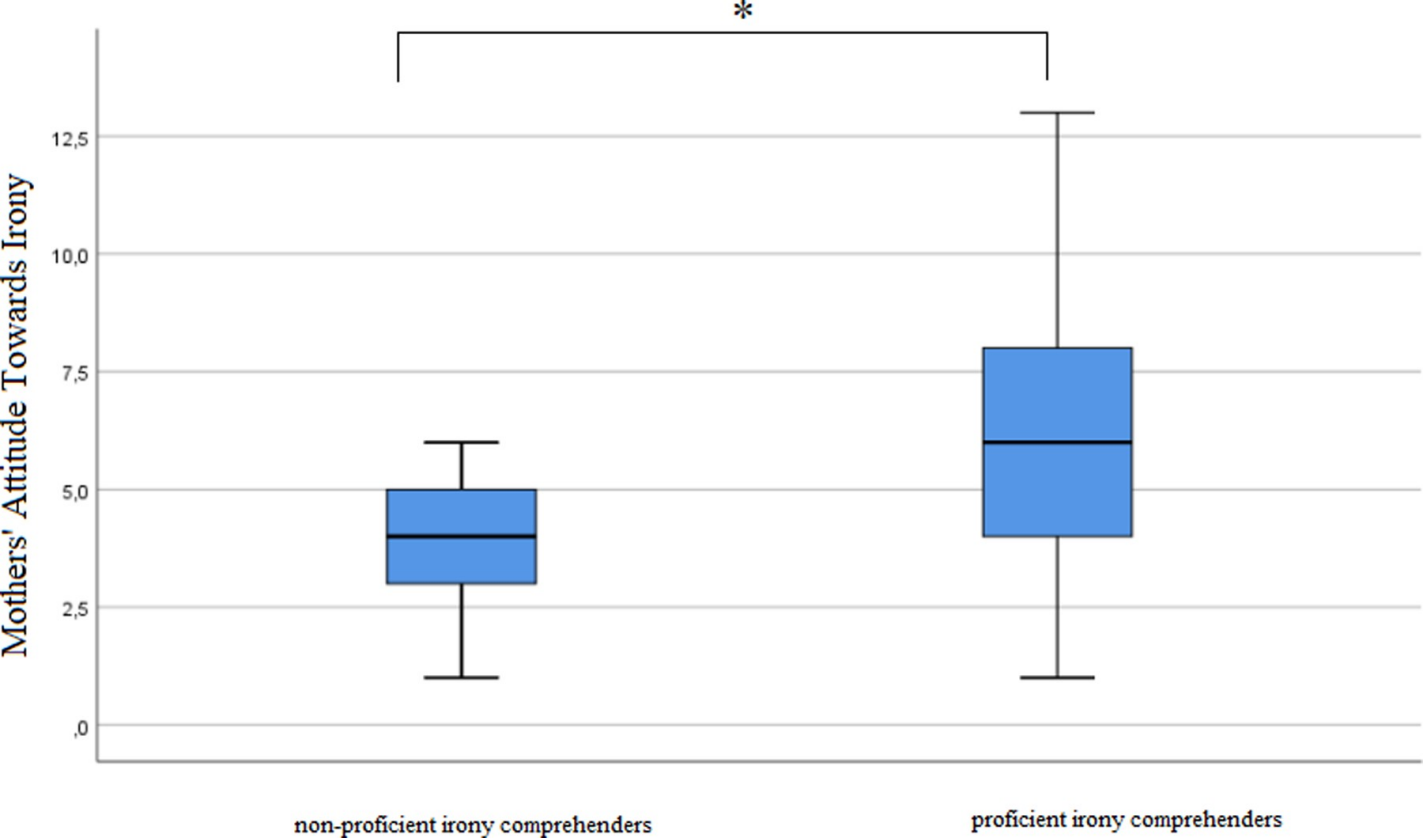
Mann-Whitney U test results displayed in box plot showing significant differences in mothers’ declared likelihood of using irony between a group of children not proficient and proficient in decoding the right meaning behind the ironic statement (*U* = 82.5, *p* = 0.034).

**Fig 3:**
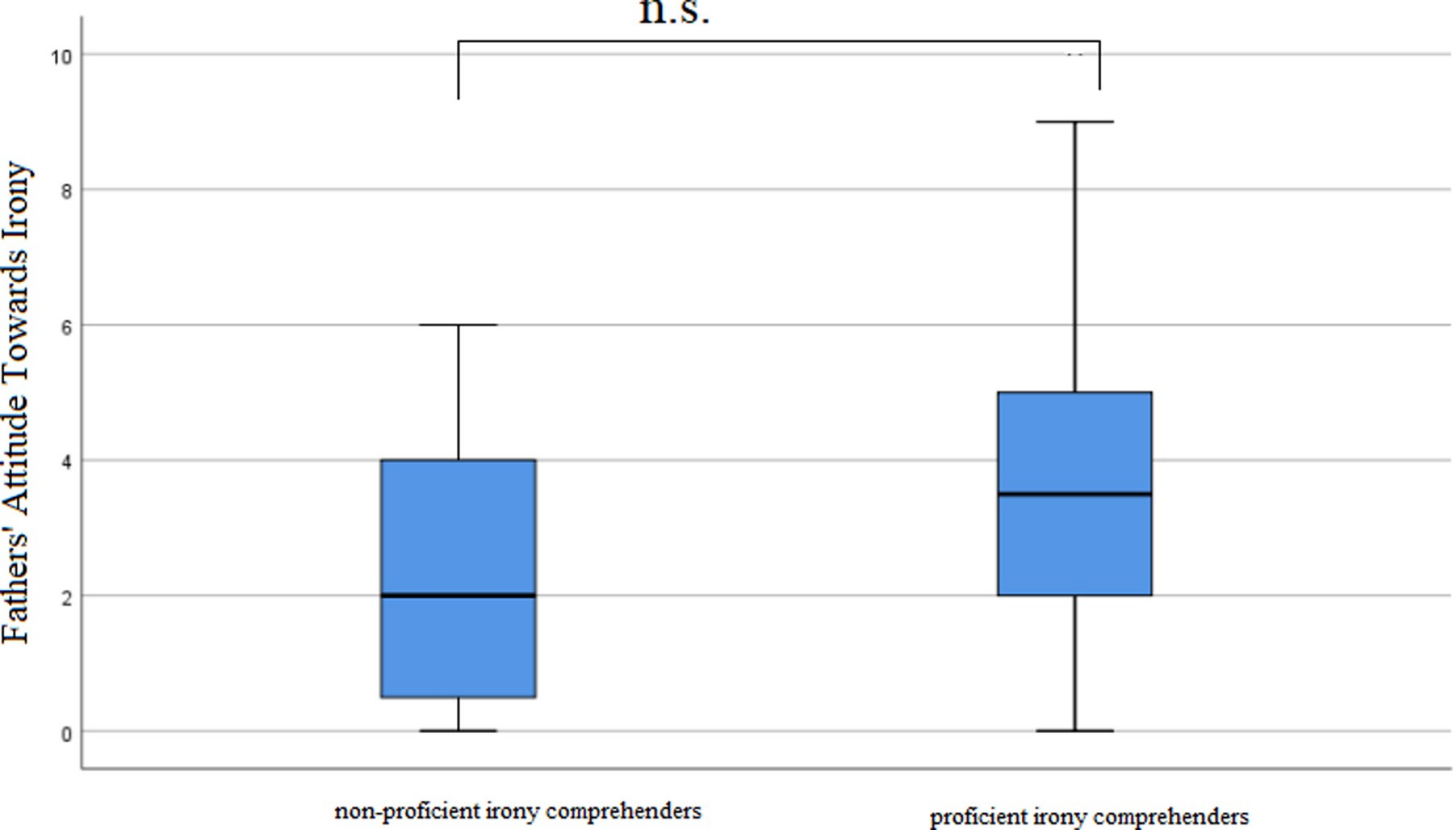
Mann-Whitney U test results displayed in box plot showing no significant differences in fathers’ declared likelihood of using irony between a group of children not proficient and proficient in decoding the right meaning behind the ironic statement (*U* = 94.5, *p* = 0.253).

Scores from parents’ attitude towards irony (both mothers’ and fathers’) and the child’s irony comprehension tasks are listed in Figs 1–3.

Measures of central tendency were computed to summarize the data for the variable. The following are the results of the analysis for mothers: *N* = 46, *M* = 5.65, *SD* = 3.049 and for fathers: *N* = 41, *M =* 3.41, *SD* = 2.674.

Due to the small range of the scores obtained by children in the Irony Comprehension Task, the score from this measure was changed into binary score. If the child answered all the comprehension questions about the meaning of the ironic statement in each story, they received the score of 1. If the child made one or more errors, the score of 0 was given. Based on the scores, children aged 8 years are either able to comprehend very simple irony or at times might misinterpret a statement or two, but we cannot treat this variable as a linear one. Hence, the variable was binary. For the binary score in irony comprehension, the measures of central tendency are as follows: *N* = 43, M = 0.79, *SD* = 0.412. Results for the Ambiguous Social Stories Task were distributed as follows: *N* = 45, *M* = 21.72, *SD* = 11.072.

Parents’ points from the Attitude Towards Irony questionnaire were summed to a total score. Version 25 of IBM SPSS Statistics was used to analyze the data.

To examine the relation between the parents’ attitude towards irony use with children and their children’s irony comprehension, we computed the Mann Whitney U-test that compared families with children who understood irony (these, who scored 1 on ICT) with those where children did not (children given the score of 0) on their parents’ score of ATI. The choice of the statistical test was motivated by the fact that the conditions for using a parametric t-test were not met. The analysis showed a significant difference between the families. Parents of children who understand irony faultlessly had significantly higher score in ATI than parents of children who do not (*U* = 72.5, *p* = 0.016).

This finding suggests that children who correctly comprehended the speaker’s intended meaning behind the ironic statement, had parents who declared that they use ironic comments towards their children more often. Such parents were also more likely to report a more positive attitude towards irony use with their children as compared to the parents of children who were not as proficient in understanding the intended meaning of ironic speakers. There were no differences found between boys and girls considering irony comprehension.

Interestingly, when comparing father’s attitudes and mother’s attitudes separately between the families, results showed that mothers’, but not fathers’ attitude towards irony use with children were significantly more positive in families where their children understood irony (*U* = 82.5, *p* = 0.034 for mothers and *U* = 94.5, *p* = 0.253 for fathers). The results of the tests are presented in Figs 1–3. High score in ATI scale indicates a more positive attitude towards irony use with children, whether a low score on ATI scale indicates a more negative attitude towards irony use with children.

No differences were found between children who were proficient and non-proficient in irony understanding in the Ambiguous Social Stories Task scores (*U* = 138.5, *p* = 0.759).

## 5. Discussion

In general, the study showed that children aged 8 comprehend the intended meaning behind the expressed ironic utterance very well. Also, we found a high individual variability among parents’ attitudes towards using irony with children. Results show that children who are proficient in understanding irony have parents that declare a more positive attitude towards irony. However, when looking at mothers and fathers separately, this only holds true for mothers.

While we did expect the difference between proficient and non-proficient irony comprehenders in their parents’ score in the ATI, surprisingly it was found that this effect is accounted for only by the mothers’ and not the fathers’ results. Considering the cultural factors of the environment the study was conducted in, we might interpret the results in two ways.

Firstly, in Poland the traditional model of family is common, where mothers spend significantly more time interacting with children and fathers usually work more hours outside of homes. Accordingly, children may be more sensitive to mothers’ comments. Past studies have shown that both parental and maternal child-raising is involved in children’s prosocial behavior [51, 52] but results referring to fathers are less compatible as results from maternal samples [53].

Another possible explanation is that the situations and particular use of the ironic comments used in the Irony Attitude Questionnaire might be more compatible with mothers’ use of irony in interactions. For instance, it included comments on generally expected behaviors (washing one’s hands before meals) or self-criticism. Recchia, Howe, Ross & Alexander [22] found that while fathers usually use irony to tease or to joke with their children, mothers’ used is more didactic-oriented: they use irony to address inappropriate behavior of the child.

While trying to interpret the result, it is also important to remember that the relationship within parent-child dyads might play a role. Children might bond more with one of the parent, which in turn can influence the learning process from each of the parents’ speech in different way. Especially that in language acquisition, it is not only the quantity of time that plays a role, but also the quality of time spent with the child.

Contrary to our expectation, no relations were found between the parents’ attitude towards irony use with children and children’s ToM or their social skills. One possible explanation may be that irony comprehension often results from the exposure to this form of language rather than processing both the literal and the figurative meaning of an ironic comment and confronting them with the possible speaker’s intentions. For example, to interpret ironic utterances, some children may use cues such as the tone of voice, nonverbal behaviors including gestures or facial expressions. Future research could further explore how gesture and non-verbal communication influences child-parent mental state talk that includes irony, humour, and other forms of figurative language.

Despite the contributions our study makes to the current discourse on parent-child mental state talk, our findings were limited due to various factors. For example, the parents who participated in the present study were mainly from Polish heritage and relatively high socio-economic status and education levels. Accordingly, future research should include samples that are representative of cultural, economic, and linguistic diversity. Also, this study focused primarily on the use of self-report measures, which may have failed to provide an accuate reflection of their the actual behavior. This study may encourage future research to combine the use of self-report measures with additional measures of Theory of Mind and other social emotional skills such as empathy, and direct observations of prosocial behaviors in naturalistic settings.

Developmentally, it is expected that pragmatic competencies will continue to develop through middle childhood until late adolescence or even beyond [54]. With varying prior results on irony comprehension, our study fits into the approach were children are credited for emerging irony comprehension, that is assuming that understanding the intended meaning behind the ironic utterance is an important developmental milestone in figurative language comprehension. To the best of our knowledge, we conducted the first study so far on the relation of parents’ use of figurative language and the children’s comprehension of it, and the results align what has been found for language acquisition in areas different than pragmatics–the role of parents is very important. Although we have not studied a possible influence from peers on irony comprehension, this certainly is a topic worth investigating on its own. Children we tested had entered elementary school a year before we tested them, so it is definitely also the school environment that might have influenced them, in addition to home environment, bus the significant shift usually happens in late elementary school.

Future research could also explore the role of culture, as well as gender in the links between parents’ use and attitudes towards irony and their children’s ToM and irony understanding. When it comes to the national culture, it is important to note that the study took place in Poland, a country that is classified as a high-context culture according to Hall’s [55] theory, that is a culture that heavily relies on implicit communication and expresses meanings indirectly to a significant extend [56,57]. It is hypothesized that the preference for indirectness in Poland has been is thought to be strengthened by its history, and namely periods of occupation and regime, which required artists and thinkers to look for ways of expression that would overcome censorship [58].

Given this need to learn how to think critically and question authority, irony was such could be considered as a useful psycholinguistic tool for social critique [59].

Although to the best of our knowledge, to date there remains a lack of quantitative studies on this topic, a large body of literary criticism concerned with irony in Polish literature and culture points to a potentially significant factor [60].

Despite its limitations, this study provides novel information that shows mothers, but not fathers, use of irony, and their attitudes towards irony influence their children’s understanding of irony. Such findings further the discourse in linguistic indirectness among parents and children’s social cognitive development and irony understanding, and has implications for educational practice that focuses on learning figurative language and social skills and the promotion of parent-child social-communication.

## 6. Conclusion

At the age of 8 years old, Polish-speaking children are able to comprehend the intended meaning behind the expressed ironic utterance in a simple picture-based story very well.

Parents vary in the irony use towards their children. Children who are proficient in understanding irony have parents that declare a more positive attitude towards irony. However, when looking at mothers and fathers separately, this only holds true for mothers.

## Supporting information

S1 Data (XLSX)

S1 Appendix (DOCX)
