# Supplementary material for: “Wonderful! We’ve just missed the bus.” – Parental use of irony and children’s irony comprehension
Source: PLoS One. 2020 Feb 21;15(2):e0228538. doi: 10.1371/journal.pone.0228538 (PMC7034895; doi:10.1371/journal.pone.0228538)
Supplement: S1 Appendix — (DOCX) [file pone.0228538.s002.docx]

Story 1

Krzyś wanted to have some juice. He asked his brother for the juice. Krzyś’s brother poured him a glass of juice. Krzyś knocked down the glass and spilt the juice over the clean tablecloth. There was a big, wet stain on the tablecloth. ‘Well done!’, said Krzyś’s brother to Krzyś.

Story 2

Krzyś is coming back from kindergarten with his mum. They want to get back home quickly. It starts raining. Krzyś and his mum are running to catch a bus. But the bus door has closed. The bus has left without them. ‘How lucky we are today’, says Krzyś’s mum

Story 3

Gosia dislikes spinach very much. She never eats it at kindergarten. There is spinach for dinner today. Gosia does not want to eat spinach. She says to her friend, ‘Well, my favorite food!’.
